# Ablation for Atrial Fibrillation in Combination With Left Atrial Appendage Closure: First Results of a Feasibility Study

**DOI:** 10.1161/JAHA.112.002212

**Published:** 2012-10-25

**Authors:** Martin J. Swaans, Martijn C. Post, Benno J.W.M. Rensing, Lucas V.A. Boersma

**Affiliations:** 1 Department of Cardiology, St. Antonius Hospital, Nieuwegein, the Netherlands

**Keywords:** atrial fibrillation, devices, prevention, stroke, vitamin K antagonists

## Abstract

**Background:**

Drug-refractory atrial fibrillation (AF) increasingly is being treated with catheter ablation. However, the long-term success rate, expressed as freedom from AF, is <50%. Therefore, vitamin K antagonists, with all their complications, remain necessary. Recently, left atrial appendage (LAA) occlusion devices were introduced as an alternative to vitamin K antagonists. Here, we investigated whether AF ablation and LAA occlusion could be a feasible and safe combination in patients with symptomatic drug-refractory AF and a CHADS_2_ score ≥1 or a contraindication for vitamin K antagonists.

**Methods and Results:**

Ablation was performed by using multielectrode catheters with phased radiofrequency energy. LAA was occluded with the Watchman device (Atritech, Inc, Plymouth, MN). Between February 2010 and February 2011, 30 patients were treated (21 male; age, 62.8±8.5 years). Median CHADS_2_ score was 2.5 (25th to 75th percentiles: 2 to 3), median CHADS-VASc score was 3 (25th to 75th percentiles: 3 to 5), 77% had prior stroke, and 27% had a contraindication for vitamin K antagonists. Median HAS-BLED score was 2 (range, 1 to 5). Successful device implantation was achieved with a median number of 1.5 devices (median diameter 24 mm [25th to 75th percentiles: 24 to 24 mm]). Total procedure time was 97 minutes (25th to 75th percentiles: 75 to 115 minutes). At 60 days, all patients met the criteria for successful sealing. Follow-up visit at 12 months showed a 30% rate of documented recurrence of AF. A repeated pulmonary vein isolation was performed successfully in 4 patients, without interference of the LAA closure device. No thromboembolic events occurred during 1-year follow-up.

**Conclusion:**

LAA occlusion with the Watchman device and AF ablation can be combined successfully and safely in a single procedure. The Watchman does not interfere with a repeated ablation.

## Introduction

Atrial fibrillation (AF) might occur in 1% to 2% of the general population, with a lifetime risk of 24% in persons >40 years of age.^1–3^ Cerebral stroke is one of the major complications of AF because of formation of atrial thrombi, especially in the left atrial appendage (LAA).^4–5^ Autopsy and echocardiography studies have shown that the LAA was the source of thrombi in >90% of the patients with nonvalvular AF.^4^ The overall annual stroke risk is 5% in patients with AF, increasing up to 15% in high-risk patients.^6^ According to the guidelines, anticoagulation should be given to prevent thromboembolic events.^1^ However, several studies such as the Euro Heart Survey showed that 28% of high-risk patients, especially elderly patients, were undertreated.^7–8^ Furthermore, vitamin K antagonists (VKA) have several disadvantages, such as (major) bleedings, nontolerance, noncompliance, interactions with some dietary components and other medications, and a narrow therapeutic range.^9–12^ The modern alternative to VKA is dabigatran. Unfortunately, dabigatran also has been associated with similar risk of major hemorrhage.^9,12^ A percutaneous mechanical obliteration or exclusion of the LAA from the systemic circulation could be an alternative. The success of such a device has been shown in a recent randomized clinical trial.^13–15^

Drug-refractory AF increasingly is being treated with catheter ablation because multiple randomized studies have shown a significantly better rhythm outcome with catheter ablation than with antiarrhythmic drug treatment.^16–18^ The long-term efficacy of catheter ablation is disappointing, with success rates <50%.^19^ The combination of LAA occlusion with catheter ablation might be an elegant way to cure or ameliorate the symptoms of AF, while at the same time reducing the risk of stroke and abolishing the need for VKA. We describe our series of LAA occlusion in combination with AF ablation in a single procedure.

## Methods

### Patient Selection

This was an open-label, nonrandomized, prospective registry. Patients ≥18 years of age with documented paroxysmal, or (longstanding) persistent, nonvalvular AF were eligible if they had an increased risk for stroke (CHADS_2_ score ≥1) or (relative) contraindication for VKA. The stroke risks according to the CHADS_2_, CHA_2_DS_2_-VASc, and the HAS-BLED scores were calculated. Before the procedure, transesophageal echocardiography (TEE) was performed to determine LAA anatomy and to exclude thrombus. All patients included in the study were fully informed about the procedure and signed a written consent form. The study was approved by the hospital's ethics committee. The procedures were performed in accordance with the hospital's ethics standards and the Helsinki Declaration of 1975 (revised in 2008).

### Procedure

#### Electrophysiological and Ablation Procedure

Electrophysiological catheter ablation procedures were performed with patients under general anesthesia. VKA was lowered before the procedure to achieve an international normalized ratio of 2.0 to 3.0. Antiarrhythmic drugs were continued up to the time of the procedure. Electrophysiological study was performed with an electrophysiological recording system (Bard, Inc, Lowell, MA) with filter settings of 100 to 500 kHz and signal amplification set at 5000. Pulmonary vein (PV) isolation was performed with the PV ablation catheter (PVAC; Medtronic/Ablation Frontiers, Inc, Carlsbad, CA). The PVAC is a 9F, over-the-wire, circular, decapolar mapping and ablation catheter with a 25-mm-diameter array at the distal tip. No additional nonfluoroscopic guiding or steering systems were used. A 7F sheath was introduced through the right femoral vein. A quadripolar catheter was introduced into the coronary sinus for pacing purposes. A standard transseptal puncture was performed with an RF Brockenbrough needle (Baylis Medical Company Inc, Montreal, Quebec, Canada) with a 12.5F-outer-diameter/9.5F-inner-diameter steerable sheath (Channel, Bard, Lowell, MA) Angiography was performed via the sheath to delineate the PVs and LAA. A single heparin bolus of 10 000 international units was administered intravenously through the sheath after transseptal puncture, and after 1 hour, a repeated bolus of 5.000 international units was administered each additional half-hour, without activated clotting time measurements.^20^ In addition to PVAC PV isolation, in some patients with longstanding persistent AF, complex-fractionated atrial electrograms ablation was performed with a multiarray septal catheter (MASC) and a multiarray ablation catheter (MAAC).^21^

#### LAA Closure

The Watchman implantation was performed immediately after the ablation procedure by using monoplane fluoroscopy and 3-dimensional TEE guidance. The initial 12.5F sheath was replaced by a 14F transseptal access sheath (Atritech, Inc, Plymouth, MN), which was positioned in the LAA. With a pigtail catheter, additional angiograms were made to determine size and shape of the LAA. This access sheath serves a conduit for the delivery catheter, which contains the device (WATCHMAN LAA Occlusion Device, Atritech, Inc, Plymouth, Minnesota, MN), a self-expanding nitinol frame with fixation barbs and a permeable polyester fabric cover. Details of the Watchman have been described elsewhere.^13–14^ Briefly, there are 5 device sizes (21, 24, 27, 30, and 33 mm) to accommodate varying LAA anatomy and size. A device size 10% to 20% larger than the largest diameter of the LAA body (as measured by angiography and TEE) was chosen, to have sufficient compression for stable positioning. The device deploys by retracting the access sheath. Before releasing it from the delivery catheter, several release criteria had to be fulfilled, including proper LAA position, no or minimal (<5 mm) residual lateral flow past the device, and a tug test for stability. If all these device release criteria were confirmed, the device was released and its position reconfirmed by angiography and TEE. Patients were discharged the next day after a chest x-ray to verify the Watchman position in the heart. VKA was started as standard of care after ablation and LAA device implantation (international normalized ratio between 2 and 3), if necessary with bridging low-molecular-weight heparin until international normalized ratio was >2.0. Aspirin, clopidogrel, or both were started if there were contraindications for VKA.

#### After the Procedure

Patients were seen in the outpatient clinic 90 days after the procedure. Before this visit, at 60 days, TEE was performed to evaluate LAA occlusion, thrombus formation, device position, and residual flow. If the echocardiographic criteria for successful sealing of the LAA were met (LAA completely sealed or a minimal residual flow [<5 mm jet] around the device), discontinuation of VKA was allowed at the discretion of the treating cardiologist. In that case, aspirin was continued indefinitely, and unless contraindicated, clopidogrel was started (75 mg daily) for 6 months. If these echocardiographic criteria for successful sealing were not met, TEE was repeated at 3 to 6 months.

#### Outcome

Successful device implantation was defined as long-term sealing of the LAA as measured by TEE 60 days after the procedure without major adverse events. Major adverse events were defined as death, stroke, systemic embolism, and major bleedings requiring invasive treatment or blood transfusion. Freedom from left atrial arrhythmias was described after a blanking period of 3 months.

### Statistical Analysis

Descriptive statistics were used to report patients' characteristics. Continuous variables with normal distribution are reported as mean±standard deviation. Median and 25th to 75th percentiles were used when normal distribution was absent. Percentages were used to report categorical variables. All statistical analyses were performed in SPSS software (SPSS Inc, version 17.0 for Windows).

## Results

### Patient Characteristics

Between February 2010 and February 2011, 30 patients (mean age, 62.8±8.5 years; 30% female) were treated. The characteristics of the patients are given in Table 1. All patients had documented AF and a CHADS_2_ score of ≥1 that necessitated the use of oral anticoagulation. The median CHADS_2_ score was 2.5 (25th to 75th percentiles: 2 to 3), median CHA_2_DS_2_-VASc-score was 3 (25th to 75th percentiles: 3 to 5), and HAS-BLED-score was 2 (25th to 75th percentiles: 1 to 3). Twenty-three patients (77%) had a history of stroke, of whom 9 (30%) had a stroke under oral anticoagulation. Eight patients (27%) had a relative contraindication for VKA that was due to bleeding or failure to achieve an adequate international normalized ratio, and 2 patients had both. All patients but 2 (93%) used VKA before the procedure. The average maximum LAA diameter was 19.4±2.2 mm, and the depth was 31.0±5.5 mm. In 27% of the patients, there was a multilobular atrial appendage.

**Table 1.** Baseline Characteristics

Number	30
Age, y	62.8±8.5
Sex, n (%)	
Male	21 (70)
Female	9 (30)
Type of AF, n (%)	
Paroxysmal AF	13 (43)
Persistent AF	12 (40)
Long-standing persistent AF	5 (17)
Median CHADS_2_	2.5 (2–3)
1	2 (7)
2	13 (43)
3	11 (37)
4	4 (13)
CHA_2_DS_2_-VASc	3 (3–5)
HAS-BLED	2 (1–3)
Anticoagulation, n (%)	
Coumadin	28 (93)
Aspirin	2 (7)
Antiarrhythmic drugs,* n (%)	
Class I	16 (53)
Class II	13 (43)
Class III	11 (37)
Class IV	2 (7)
Upstream therapy, n (%)	
Statin	15 (50)
ACE-I/ARBs	17 (57)
Indication, n (%)	
Bleeding with VKA	8 (20)†
Stroke with VKA	9 (30)†
Patient preference	15 (50)
LAA	
LAA width, mm	19.4±2.2
LAA length, mm	31.0±5.5
Multilobular, n (%)	8 (27)

All data are presented as mean ± standard deviation, n (%), or median (25th–75th percentiles). AF indicates atrial fibrillation; ACE-I, angiotensin-converting enzyme inhibitors; ARBs, angiotensin II receptor blockers; VKA, vitamin K antagonist; and LAA, left atrial appendage.

* According to the Vaughan-Williams classification.

† Two patients had a history of both bleeding and a stroke under VKA therapy as the indication for the Watchman device.

### Procedural and In-Hospital Results

The median total procedure time was 97.3 minutes (25th to 75th percentiles: 75 to 115 minutes), including a median 38 minutes (25th to 75th percentiles: 30 to 51 minutes) for LAA occlusion. Twenty-two patients (73%) were treated with only PV isolation with PVAC ablation, whereas in the other 8 patients, additional complex-fractionated atrial electrograms ablation was performed with the MASC and MAAC. The median size of the LAA device was 24 mm (25th to 75th percentiles: 24 to 24 mm). A median of 1.5 devices per patient (25th to 75th percentiles: 1 to 2) was required to select the right size for optimal LAA closure. At the end of the procedure, 3 patients had minimal residual flow (flow ≤5 mm); in all other cases there was complete closure of the LAA. There were no major and only 3 minor periprocedural complications: One patient developed a small tongue hematoma after the procedure, probably because of manipulation of the TEE probe, and 2 patients developed a small groin hematoma. None required intervention. All patients were discharged the next day (Table 2).

**Table 2.** Periprocedural Characteristics

Procedure, n (%)	
PVAC	22 (73)
PVAC/MASC/MAAC	8 (27)
Device	
Number	1.5 (1–2)
Size, mm	24 (24–24)
Size, n (%)	
21	5 (17)
24	18 (60)
27	6 (20)
30	1 (3)
Total procedure time, min	97.3 (75–115)
LAA closure, min	38 (30–51)
Total fluoroscopy time, min	15.5 (13–19)
Complications, n (%)	
Pericardial effusion	0 (0)
Air embolism	0 (0)
Major bleeding	0 (0)
Minor bleeding	3 (10)
TEE, n (%)	
Successful implantation	30 (100)
Minimal residual flow	3 (10)
Hospitalization, days	2 (2–2)

All data are presented as mean ± standard deviation, n (%), or median (25th–75th percentiles). PVAC indicates pulmonary vein ablation catheter; MASC, multiarray septal catheter; MAAC, multiarray ablation catheter; LAA, left atrial appendage; and TEE, transesophageal echocardiography.

### Follow-Up Results

#### LAA Closure

TEE was performed after 60 days and showed successful sealing of the LAA in all patients. Twenty-three patients (77%) had complete sealing of the LAA, and in 7 patients (23%) there was minimal residual flow (jet diameter <5 mm). In these patients with minimal residual flow, repeated TEE at 6 months showed complete sealing in an additional 5 patients, resulting in complete occlusion in 93% of patients. In 17 of the 30 patients, the VKA was discontinued and aspirin started at 60-day follow-up. In 23 patients (77%), the VKA was discontinued at 12-month follow-up (Table 3). Device embolization was the rationale to continue VKA in one patient. One patient had dense spontaneous contrast in the left atrium, and another patient developed pulmonary embolism, both requiring resumption of the VKA therapy. Finally, in 4 patients, continuation of VKA was the treating cardiologist's preference on the basis of recurrent or persistent AF.

**Table 3.** Follow-Up Characteristics at 12 Months

Number	30
TEE, n (%)	
Minimal residual flow	2 (7)
Device embolization	1 (3)
Thrombus on device	0 (0)
Freedom from left atrial arrhythmias	21 (70)
Redo ablation	4 (13)
Surgical (Mini) Maze and LAA amputation	3 (10)
Coumadin, n (%)	7 (23)
Complications during follow-up, n (%)	
Death	0 (0)
Stroke or transient ischemic attack	0 (0)
Major bleeding	3 (10)*
Minor bleeding	0 (0)

All data are presented as mean ± standard deviation, n (%), or median (25th–75th percentiles). TEE indicates transesophageal echocardiography.

* All patients were still on oral anticoagulation during bleeding.

#### Safety and Bleeding

None of the patients had thrombus formation on the surface of the device. In 1 patient, at TEE follow-up at 3 months, the LAA device was found to have asymptomatically dislocated to the abdominal aorta. The device was retrieved successfully by means of a percutaneous transfemoral procedure. At 12-month follow-up, no thromboembolic events had occurred. Three patients had a severe bleeding event. These bleedings were not procedural or device related. One patient (HAS-BLED of 1) developed hematuria requiring bladder flushing, 1 patient (HAS-BLED of 2) with Rendu-Osler Weber disease developed nose bleedings and melena requiring transfusion, and 1 patient (HAS-BLED of 4) had intestinal bleeding, unmasking colorectal malignant neoplasm requiring a hemicolectomy. As mentioned previously, 3 patients had a procedure-related minor bleeding. All bleedings occurred within the 60 days after ablation, with all patients still on VKA, given that discontinuation was not allowed before the echocardiographic criteria for complete sealing were met on the control TEE.

#### Left Atrial Arrhythmia Recurrence

After a blanking period of 3 months, 21 of the 30 patients (70%) were asymptomatic and did not have any documented AF on ECG recordings during regular 12-month follow-up. Of the 9 patients who had a clinical recurrence, 6 were treated for persistent AF and 3 for paroxysmal AF. No systematic Holter analysis was performed for AF detection. Four redo ablations were carried out in the remaining 9 patients who were not free of AF during 12-month follow-up. The LAA device was not affected and did not interfere with the redo catheter ablation. During redo ablations, no triggers from the LAA were observed. Three patients became asymptomatic, and no AF recurrence has been recorded so far. The fourth patient who underwent a redo ablation is still in the blanking period.

Three patients who were not free of AF were treated surgically with a minimally invasive MAZE procedure. One of them still has persistent AF. Recurrent AF was accepted in the remaining 2 patients.

## Discussion

In this study, we describe LAA occlusion in combination with AF ablation in a single procedure in patients with nonvalvular AF with a moderate to severe risk of stroke or contraindication for VKA. Our results indicate that a high procedural success rate with a relatively low complication rate can be obtained, with satisfactory mid-term follow-up results.

### Procedural Success

The PROTECT AF (WATCHMAN Left Atrial Appendage System for Embolic PROTECTion in Patients with Atrial Fibrillation) trial was the first multicenter noninferiority trial that randomized 707 patients with nonvalvular AF in a 2:1 schema to either the Watchman device as standalone therapy or (conventional) treatment with VKA. The device was implanted successfully in 88% of the patients assigned to the intervention group. In this group, 86% met the TEE criteria for adequate sealing at 60 days, and 92% met the criteria at 6 months.^13–14^ In our present study, the device was implanted successfully in all patients. At 60 days, TEE showed successful sealing of the LAA in all patients according to the criteria of the PROTECT AF trial, although there was a minimal residual flow in 7 patients (23%). At 6-month follow-up, complete sealing was observed in 93% according to TEE. This is comparable to the PROTECT AF trial. Viles-Gonzalez et al^22^ have shown that residual flow around the device is not associated with an increased risk of thromboembolic events. Although these results must be handled with caution, our study also suggests no increased risk of thromboembolic events in patients with minimal residual flow during 1-year follow-up, confirming the results of the study performed by Viles-Gonzalez.

### Safety

Safety endpoints in the PROTECT AF trial were major bleeding, pericardial effusion, and device embolization. These primary safety endpoints were more frequent in the intervention group than in the control group (7.4% per 100 patient-years versus 4.4% per 100 patient-years; relative risk 1.69 [95% confidence interval, 1.01 to 3.19]). The majority of these were periprocedural, predominantly caused by pericardial effusion and procedural stroke related to air embolism due to inexperience of the operator with transseptal punctures and large sheaths. Indeed, the recently published data from the Continued Access Registry showed a significant decrease in procedural events, from 7.7% in the first halve of the PROTECT AF to 5.5% in the second half and 3.7% in the Continued Access Registry. Our present study was performed in a high-volume cardiology center by an electrophysiologist and interventionalist who were experienced in left atrial procedures. Despite the fact that the implantation was combined with an ablation, none of the patients had pericardial effusion or a procedure-related stroke, which underlines the importance of operator experience. In this setting, LAA occlusion can be performed safely. Directly after the procedure, there were 3 (10%) minor bleedings. During the 6-month follow-up, there were 1 device embolization and 3 nonprocedural major bleedings in patients still using VKA. One of the disadvantages of combining a Watchman implantation with catheter ablation for AF is that patients are required to be on VKA for at least the first 3 months after the procedure. In patients for whom VKA is contraindicated, this could pose a challenge with regard to postprocedural stroke protection. The use of low-molecular-weight heparin for a shorter period of time or the new-generation factor X antagonists might be an alternative, although further studies are needed to determine the role of these drugs in ablation and Watchman implantation.

### Efficacy

#### Ablation Therapy for AF

Previously, single-procedure ablation studies with PVAC/MASC/MAAC, also from our group, in different types of AF showed mean success rates of 50% to 60% after 1-year follow-up.^20–21,23–26^ The Bordeaux group recently published results indicating 29% freedom from AF after 5-year follow-up in a mixed population of paroxysmal and nonparoxysmal AF.^19^ Although we did not systematically perform Holter monitoring, 70% of patients became asymptomatic and had no AF documented on ECG during 12-month follow-up. In prior studies from our group, the procedure and fluoroscopy times were 86±27 and 20±9 minutes, respectively, for PVAC alone and 112±32 and 21±10 minutes for PVAC/MASC/MAAC procedures.^21,27^ This is similar to the procedure times and fluoroscopy times described for the ablation part of the procedure in the present study.

Di Biase et al have shown that in a selected patient group with recurrent AF after an initial ablation, only 27% showed foci arising from the LAA.^28^ The optimal treatment was found to be complete circumferential LAA isolation. Because the Watchman device lies within the LAA itself, this should not preclude a strategy of ostial isolation of the LAA. Moreover, this patient group might especially benefit from LAA closure because electrical isolation of the LAA could lead to LAA stasis even during sinus rhythm, making it more thrombogenic. In patients for whom AF ablation and LAA closure is considered, one could first perform an electrophysiological study with pharmacological challenges to detect and ablate possible LAA triggers before implantation of the Watchman device.

#### Stroke Prevention in the Combined Approach

The composite primary efficacy endpoint of the PROTECT AF study was freedom from all stroke, cardiovascular death, and systemic embolization. In an intention-to-treat noninferiority analysis at 1065 patient-years of follow-up, the event-free probability was better in the device group (3.0% versus 4.9% per 100 patient-years; relative risk 0.62 [95% confidence interval, 0.35 to 1.25]) and met noninferiority criteria. The event rate of all ischemic and hemorrhagic strokes was lower in the intervention group than in controls (2.3% versus 3.2% per 100 patient-years; relative risk 0.71 [95% confidence interval, 0.35 to 1.64]), and all-cause mortality rate was lower in the intervention group (3.0% versus 4.8% per 100 patient-years; relative risk 0.62 [95% confidence interval, 0.34 to 1.24]). Our population had a median CHADS_2_ score of 2.5. This score is associated with an estimated annual stroke risk of 4.0% to 5.9%.^29^ During a follow-up of 12 months, none of our patients developed a thromboembolic complication, although only 30% had a documented AF recurrence, and 23% were still using VKA. The disappointing long-term efficacy of AF ablation, with success rates <50%, might be another compelling advantage of combining ablation and LAA closure.^19^ Our data suggest that the combination of AF ablation and LAA closure works well to reduce stroke risk from 2 ends. This would be especially helpful in patients with AF and a prior stroke or high stroke risk; patients with a contraindication to VKA, prior intracranial hemorrhage, or a high bleeding risk; and patients with an anticipated reduced efficacy of ablation alone. The combination of PV isolation with LAA closure in a single procedure also reduces the need for, and associated risk of, a repeated left atrial intervention and transseptal puncture should LAA closure become desirable during follow-up. High-volume randomized clinical trials would be needed to determine the benefit and cost-effectiveness of this combined intervention strategy.

Finally, novel oral anticoagulants (NOACs) like dabigatran, rivaroxaban, and apixaban have been introduced in large randomized clinical trials.^30–32^ They were found to be at least noninferior to VKA, with a lower rate of intracranial bleeding as the most important benefit. Whether these expensive NOACs will prove their value in real-life clinical practice remains to be determined. The data on periprocedural use of NOACs for ablation therapy are not yet conclusive to support their noninferiority to VKA. There are as yet no data from a head-to-head comparison of NOACs against LAA closure devices. Moreover, in patients with prior bleeding complications or a high bleeding risk, it could still be unattractive to switch to NOACs.

### Limitations

Our study has several limitations. First, the number of patients is limited, and follow-up time was relatively short. Second, it is an observational study and did not compare the percutaneous technique to conventional treatment. However, this was beyond the scope of our feasibility study. Third, even though the follow-up TEE at 60 days after the procedure showed that the criteria for successful sealing of the LAA were met in all patients and discontinuation of VKA was allowed, VKA had not been discontinued at the discretion of the treating cardiologist in 7 patients (23%), which potentially could lead to overestimation of the efficacy of the Watchman. The addition of LAA closure to an ablation procedure requires general anesthesia because of continuous TEE monitoring, which could potentially lead to more complications. Another limitation is that we did not use systematic Holter monitoring to detect freedom from AF, which could have led to an overestimation of the ablation efficacy. We did not perform a risk–benefit ratio or cost–benefit ratio of this procedure. More data on this topic could become available from a second prospective, randomized, multicenter trial (Prospective Randomized EVAluation of the Watchman LAA Closure Device in Patients with Atrial Fibrillation Versus Long Term Warfain Therapy [PREVAIL] trial) that has started in the United States of America to provide additional information on the safety and efficacy of the Watchman LAA closure; this trial also is evaluating the performance of the device in patients with AF for whom long-term VKA is contraindicated.

## Conclusion

In this “proof-of-concept” study in patients with nonvalvular AF with a moderate to severe risk of stroke or contraindication for VKA, we show for the first time that the combination of ablation and LAA occlusion with the Watchman device was performed successfully and safely and did not interfere with repeat PV isolation.
